# Ribosomal multilocus sequence typing: universal characterization of bacteria from domain to strain

**DOI:** 10.1099/mic.0.055459-0

**Published:** 2012-04

**Authors:** Keith A. Jolley, Carly M. Bliss, Julia S. Bennett, Holly B. Bratcher, Carina Brehony, Frances M. Colles, Helen Wimalarathna, Odile B. Harrison, Samuel K. Sheppard, Alison J. Cody, Martin C. J. Maiden

**Affiliations:** Department of Zoology, University of Oxford, Oxford, UK

## Abstract

No single genealogical reconstruction or typing method currently encompasses all levels of bacterial diversity, from domain to strain. We propose ribosomal multilocus sequence typing (rMLST), an approach which indexes variation of the 53 genes encoding the bacterial ribosome protein subunits (*rps* genes), as a means of integrating microbial genealogy and typing. As with multilocus sequence typing (MLST), rMLST employs curated reference sequences to identify gene variants efficiently and rapidly. The *rps* loci are ideal targets for a universal characterization scheme as they are: (i) present in all bacteria; (ii) distributed around the chromosome; and (iii) encode proteins which are under stabilizing selection for functional conservation. Collectively, the *rps* loci exhibit variation that resolves bacteria into groups at all taxonomic and most typing levels, providing significantly more resolution than 16S small subunit rRNA gene phylogenies. A web-accessible expandable database, comprising whole-genome data from more than 1900 bacterial isolates, including 28 draft genomes assembled *de novo* from the European Bioinformatics Institute (EBI) sequence read archive, has been assembled. The *rps* gene variation catalogued in this database permits rapid and computationally non-intensive identification of the phylogenetic position of any bacterial sequence at the domain, phylum, class, order, family, genus, species and strain levels. The groupings generated with rMLST data are consistent with current nomenclature schemes and independent of the clustering algorithm used. This approach is applicable to the other domains of life, potentially providing a rational and universal approach to the classification of life that is based on one of its fundamental features, the translation mechanism.

## Introduction

Since its inception as a science, microbiology has depended on the reproducible classification of bacteria into types that can be grouped systematically. The association of particular bacterial groups with specific activities, initially used to determine microbial disease aetiology, has been widely successful in identifying and investigating the activities of particular bacteria throughout the biosphere. A variety of data have been used to categorize bacteria, in a number of cases governed by formal schemes such as the International Code of Nomenclature of Bacteria (Lapage *et al.*, 1992); however, the great diversity of the bacterial domain (Krieg *et al.*, 2005) has, to date, prevented the development of a single characterization scheme that can be readily applied to all bacteria at all levels of relatedness. Whilst practical means of assigning bacterial isolates to particular taxa exist, there remains debate concerning the biological and evolutionary processes that lead to the generation and maintenance of the clusters of related organisms that form the basis of these formally defined taxa (Doolittle & Zhaxybayeva, 2009). Irrespective of these debates on the nature of bacterial species, the recent widespread application of very-high-throughput sequencing technologies to bacterial populations and communities has greatly increased the necessity for a genealogical method that can work at different taxonomic levels and which can be used to assist definitions of nomenclature (Achtman & Wagner, 2008).

Genotypic methods became increasingly important in bacterial characterization throughout the 20th century, complementing, and to an extent replacing, the phenotypic methods originally employed (Stackebrandt *et al.*, 2002). A major step was the introduction of small subunit (16S) ribosomal RNA sequences into bacterial systematics, which provided a general framework for categorizing bacteria based on evolutionary relationships, thus representing a ‘natural’ classification system (Woese, 1987). The genes encoding the 16S rRNA are effective for classification as they are universal, easily sequenced, and conserved. They have been widely employed in bacterial systematic and evolutionary studies, playing a seminal role in establishing the place of the bacteria in the three domains of life (Woese *et al.*, 1990), and being widely employed in the examination of bacterial pathogens, including in diagnostic applications (Clarridge, 2004). Sequence analysis of the 16S rRNA gene has also been used extensively in the examination of microbial communities, such as the human microbiome (Nelson *et al.*, 2010) and environmental communities (Lim *et al.*, 1993; Gray & Herwig, 1996; Gilbert & Dupont, 2011). While 16S rRNA gene phylogenies provide a general framework, they are unable to provide unification between taxonomic and typing schemes, as many bacteria with distinct properties share identical or very similar 16S rRNA gene sequences (Stackebrandt *et al.*, 2002). This is particularly the case with a number of pathogenic bacteria, where the reliable classification of closely related bacterial specimens into distinct types with particular pathogenic properties is essential for reliable diagnosis and epidemiology (van Belkum *et al.*, 2007).

The recognition that horizontal genetic exchange is widespread among bacteria (Smith *et al.*, 1991) established the necessity of employing multiple loci in characterization schemes, as frequent recombination reassorts loci, consequently breaking down phylogenetic congruence within bacterial genomes (Holmes *et al.*, 1999). Techniques such as multilocus sequence typing (MLST) (Maiden *et al.*, 1998), which indexes sequence variation at housekeeping loci, have provided a means of reliably identifying the relationships among related organisms, and have been suggested as a basis for a novel approach to microbial species identification (Gevers *et al.*, 2005). MLST, however, which typically analyses six to eight genetic loci (Maiden, 2006), does not always provide sufficient resolution among very closely related bacteria (Achtman, 2008). Furthermore, because of the diversity of bacterial metabolism across the domain, and even among quite closely related organisms, each MLST scheme has to be developed for a particular group of related bacteria. MLST schemes are therefore usually limited to bacteria belonging to the same genus, and even within a given genus, several distinct MLST schemes may be required (Maiden, 2006). Thus, although providing a general approach for both bacterial genealogy and typing, MLST and the related method of multilocus sequence analysis (MLSA) (Gevers *et al.*, 2005) do not provide a practical combined taxonomic and typing approach at all levels of bacterial diversity.

The recent rapid expansion of nucleotide sequencing capacity with ‘next-generation’ sequencing technology has greatly increased the number of bacterial genomes available for analysis (Medini *et al.*, 2008), with numbers expanding rapidly (Chain *et al.*, 2009). This enables a comparative analysis of the genetic variation at shared loci across the whole domain. Here we use a new database platform, Bacterial Isolate Genome Sequence Database (BIGSdb; Jolley & Maiden, 2010), to implement a combined taxonomic and typing approach for the whole domain Bacteria that provides resolution down to the strain or subspecies level, by indexing the sequences of ribosomal protein-encoding genes in a curated MLST scheme, ribosomal MLST (rMLST). Use of the scheme will enable rapid species and strain identification directly from whole-genome data generated by parallel sequencing techniques (Supplementary Fig. S1).

## Methods

### 

#### Genomes.

Whole-genome data from 1902 strains representing the entire bacterial domain were downloaded from the Integrated Microbial Genomes (IMG) database (Markowitz *et al.*, 2010). These were uploaded to a BIGSdb database, along with taxonomic and provenance data where available. In addition, Illumina whole-genome data for 28 isolates of Pneumococcal Molecular Epidemiology Network 1 (PMEN1) *Streptococcus pneumoniae* (Croucher *et al.*, 2011) were extracted from the European Bioinformatics Institute (EBI) sequence read archive. The Velvet *de novo* assembler (Zerbino, 2010; Zerbino & Birney, 2008), shuffle and optimization scripts were used to create contigs using optimal parameters, with kmer lengths between 21 and 51 bp, with no scaffolding. The assembled contigs were uploaded into the BIGSdb database.

#### Identification and tagging of *rps* genes.

A reference gene BIGSdb database was set up to contain the entire known sequence diversity of the ribosomal protein genes, and this was seeded with data from a few species manually retrieved from IMG and GenBank annotations. The ribosomal protein genes were then tagged in the database by an iterative process of identification by blastn and tblastx (Altschul *et al.*, 1997) searches of the whole genome data against previously defined alleles at progressively lower stringency. Initial blastn parameters had a cut-off of 70 % identity over 50 % of the alignment length and a word size of 15. Each unique sequence was defined with an arbitrary allele number. After definition of new alleles, any genomes without alleles assigned were scanned again with the same parameters. This was repeated until no further matches were found. At this point, the stringency was reduced by 5 % identity and the process repeated until no further matches were identified down to a stringency of 50 % identity. At this point, tblastx searches were performed using the same iterative process. When no further matches were identified, extra seeding of the allele database was achieved by an automated Perl script that retrieved alleles from the National Center for Biotechnology Information (NCBI) Entrez Gene database for species with missing data, and the iterative blast scanning was repeated, starting at the initial stringency settings. Finally, allele sequences were cleaned to ensure that only in-frame sequences without internal stop codons were included and the locus position definitions fixed so that the sequences began at common start sites, where possible. Since allele identification was performed by an iterative process of matching to previously defined sequences, periodic checks of new alleles were made by blasting against GenBank to ensure that they did not match other genes. Tagging of the 16S rRNA gene was achieved following initial seeding of the definition database with only a single sequence and blastn matching.

#### Phylogenetic analysis.

Phylogenetic trees were generated by exporting ribosomal protein gene sequences from the strain database as an XMFA file containing each locus as an aligned block. clonalframe analysis was performed for single-genus datasets using clonalframe version 1.2 (Didelot & Falush, 2007) with default parameters. For larger datasets, up to the entire bacterial domain, the XMFA file was converted to an aligned concatenated sequence for neighbour-joining tree analysis using mega version 5 (Kumar *et al.*, 2008), with ambiguous positions removed for each sequence pair. Split decomposition analysis was performed using splitstree version 4 (Huson & Bryant, 2006) for species level datasets. For the *S. pneumoniae* tree, the *rpmG* locus was removed from the analysis since three paralogues were identified. No other paralogous loci were removed from any other tree. Interactive Tree of Life (iTOL) (Letunic & Bork, 2011) was used to visualize large trees.

To assess congruence, maximum-likelihood (ML) phylogenetic trees were constructed using paup version 4 beta 10 (Swofford, 1998) on finished genomes from the entire class Bacilli (*n* = 144). ML trees for 10 ribosomal protein genes (*rpsB*, *rpsC*, *rpsD*, *rpsE*, *rpsG*, *rpsI*, *rpsK*, *rpsL*, *rpsP* and *rpsT*) with sizes between 400 and 1100 bp were computed and compared using the Shimodaira–Hasegawa test, which determines whether significant differences occur among the tree topologies (differences in log likelihood, Δ−ln L). Randomization tests were then performed (Holmes *et al.*, 1999), where the Δ−ln L values for each of the genes were compared with the equivalent values computed for 200 random trees created from each gene. This analysis was carried out on finished genomes from the entire class Bacilli (*n* = 144).

Overall mean distances (p-distance) were calculated for each locus by taxonomic class. Sequences were exported from BIGSdb, aligned by codon [using the Multiple Sequence Comparison by Log-Expectation (muscle) tool with default parameters (Edgar, 2004)], and the number of base differences per site was averaged over all sequence pairs. All ambiguous positions were removed for each sequence pair. A codon-based test of positive selection was also performed for each locus by taxonomic class using the Nei–Gojobori method (Nei & Gojobori, 1986). The probability of rejecting the null hypothesis of strict neutrality (d_N_ = d_S_) was tested in favour of the alternative hypothesis (d_N_>d_S_). Again, all ambiguous positions were removed for each sequence pair. These analyses were performed in mega5 (Tamura *et al.*, 2011). The Hunter–Gaston discriminatory index (Hunter, 1990; Hunter & Gaston, 1988) was determined for each locus for the entire dataset, the genus *Bacillus*, the monomorphic species *Bacillus anthracis* and *Yersinia pestis*, and for the highly recombinagenic species *Helicobacter pylori*. This analysis was performed using the web tool at http://insilico.ehu.es/mini_tools/discriminatory_power/.

## Results

### Uploading of genome sequences

Genome data for 1902 bacterial isolates were downloaded from the IMG database (Markowitz *et al.*, 2010) and uploaded to a BIGSdb database (available at http://rmlst.org/). These were classified as either finished (982, 51.6 %), draft (911, 47.9 %) or permanent draft (9, 0.5 %). Finished genomes had a mean of 1.7 contigs per genome, whereas draft and permanent draft genomes were together represented by 215.5 contigs per genome (Table 1). The dataset did not represent an unbiased sampling of bacterial diversity but was limited to taxa for which whole-genome data were available.

**Table 1.** Tagging status of the 53 rMLST loci used in analysis

Parameter	IMG genome status			*S. pneumoniae* velvet assemblies
	Draft	Complete	Total	
Strains uploaded	920	982	1902	28
Mean contigs per strain	215.5	1.7	105.1	354.6
Strains with all rMLST loci tagged	499	674	1173	28
Average complete rMLST genes per strain*	48.94 (53)	52.59 (53)	50.82 (53)	52.6 (53)
Average partial rMLST genes per strain*	1.077 (0)	0.003 (0)	0.523 (0)	0.39 (0)
Average missing rMLST genes per strain*	2.990 (0)	0.404 (0)	1.655 (0)	0.00 (0)
Average internal stop codons per strain*	0.702 (0)	0.329 (0)	0.509 (0)	1.00 (1)

* Averages: main values are mean; modal values are in parentheses.

### *De novo* assembly of *S. pneumoniae* genomes

Genome data for 28 *S. pneumoniae* isolates were assembled from short-read archive data into contigs by velvet assembly. The assemblies varied in hash length from 21 to 37 bp, and the total number of bases in contigs ranged from 1 736 028 to 2 198 465. Values for the length N for which 50 % of all bases in the sequences are in a contig of at least the given length (N50) ranged from 1451 to 47 198 (Supplementary Table S1). These data were uploaded into the BIGSdb database and the quality of assemblies was assessed by blast searches of *rps* loci. This identified complete coding sequences for 52 ribosomal protein genes from assemblies of 24 of the 28 isolates, confirming that these represented ‘high-quality draft genomes’. Multiple stop codons were identified in the *rplX* locus of sequence assemblies from all 28 isolates, a finding in common with 24 of the 29 *S. pneumoniae* genomes uploaded from the IMG database, indicating that the sequence is a pseudogene in this species. Incomplete *rps* sequences were identified at a single, but different locus, for each of three isolates, and sequences for eight loci were incomplete for a fourth strain. Optimized velvet parameters thus provided full coding sequence for 1445 of a potential 1456 (99.2 %) ribosomal genes, from these assemblies.

### Locus identification and curation

The majority of the 53 *rps* genes were identified in each of the bacterial genome sequences analysed, by successive blast searches. This enabled complete *rps* gene profiles (53 loci) to be generated for 1173 genomes (61.7 %), although a few genes were apparently missing in some datasets or were present as partial coding sequences that were situated at the ends of contigs. These missing or partial genes were mainly found in genomes with ‘draft’ status, as designated in the IMG database (Table 1). For the complete genomes an overall mean of 52.59 out of 53 (99.2 %) rMLST loci were identified as full sequences. Most of the remainder were completely missing (no partial sequence identified). Some sequences matched at high identity to complete alleles had nucleotide substitutions that resulted in frameshifts, resulting in internal stop codons (a mean occurrence of 0.329 out of 53 rMLST loci in the complete genomes). In many cases, these appeared to be the result of sequencing errors in homopolymeric tracts, often in genomes sequenced using 454 technology. A total of 674 (68.6 %) complete genomes were tagged at all rMLST loci. As there are 53 loci used in the rMLST scheme, the absence of a gene or its presence as a pseudogene did not affect the accuracy of the approach. In some cases multiple loci were found for particular *rps* genes, and ultimately active curation will be needed for the *rps* loci schemes for the various groups of bacteria. This could be best achieved by specialist curators with knowledge of particular groups of organisms, as is currently done for MLST schemes.

### Species discrimination

**Fig. 1.**
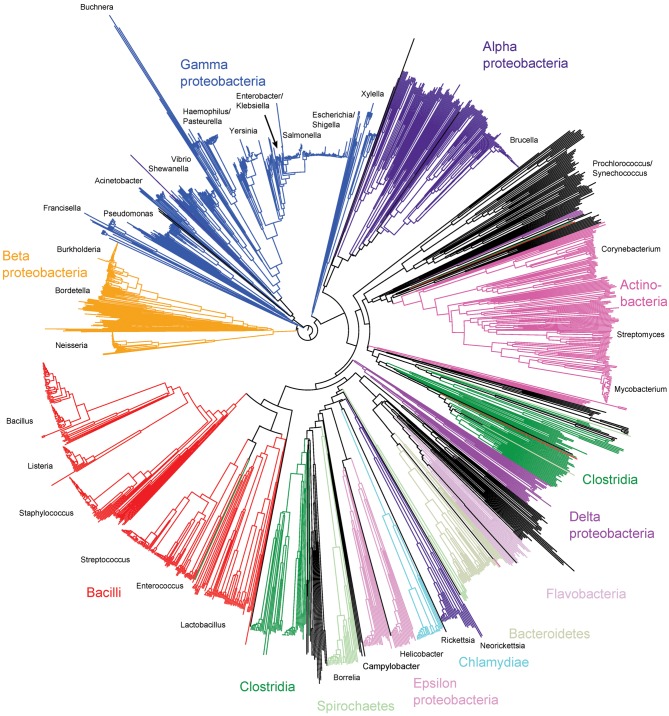
Neighbour-joining tree of the entire bacterial domain reconstructed from concatenated ribosomal protein gene sequences. The analysis involved 1565 sequences from genomes with at least 52 tagged ribosomal protein genes. Subspecies-level resolution is evident.

**Fig. 2.**
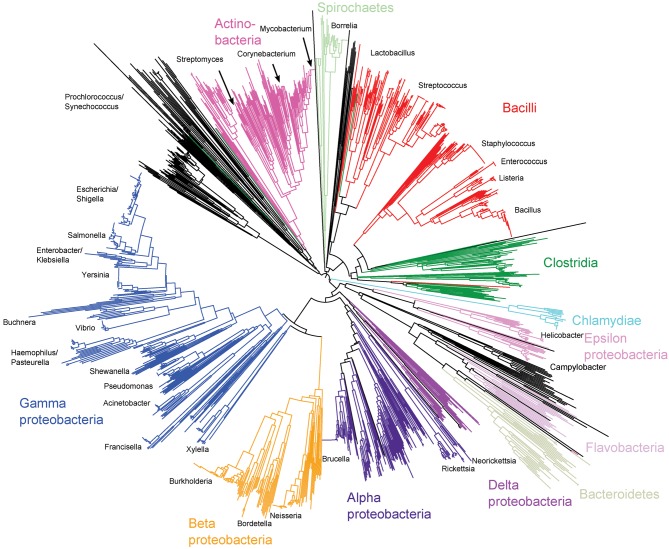
Neighbour-joining tree of the entire bacterial domain reconstructed from 16S rRNA gene sequences extracted from whole-genome data of 1663 strains.

**Fig. 3.**
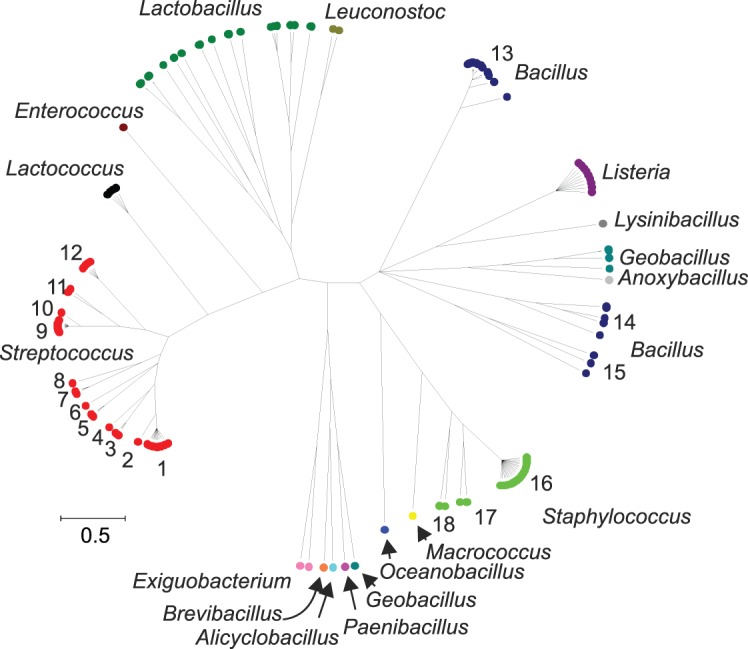
clonalframe tree of the class Bacilli using ribosomal protein gene sequences. Two independent, converged runs were merged and a 95 % consensus tree generated. Only finished genomes with 53 ribosomal protein genes identified and tagged were included in the analysis (*n* = 144). Key: 1, *Streptococcus pyogenes*; 2, *Streptococcus dysgalactiae*; 3, *Streptococcus equi*/*Streptococcus zooepidemicus*; 4, *Streptococcus. uberis*; 5, *Streptococcus agalactiae*; 6, *Streptococcus mutans*; 7, *Streptococcus thermophilus*; 8, *Streptococcus gallolyticus*; 9, *S. pneumoniae*; 10, *Streptococcus mitis*; 11, *Streptococcus gordonii*/*Streptococcus sanguinis*; 12, *Streptococcus suis*; 13, *B. anthracis*/*B. cereus*/*B. weihenstephanensis*; 14, *B. licheniformis*/*B. amyloliquefaciens*/*B. subtilis*/*B. pumilus*; 15, *B. halodurans*/*B. pseudofirmus*/*B. clausii*; 16, *Staphylococcus aureus*; 17, *Staphylococcus epidermidis*/*Staphylococcus haemolyticus*; 18, *Staphylococcus carnosus*/*Staphylococcus saprophyticus*.

Ribosomal protein genes from bacteria belonging to 452 genera were identified in the database and their contig locations tagged. Neighbour-joining trees were constructed from the concatenated sequences of 1565 isolates that had sequences determined for at least 52 of the 53 loci. These trees exhibited clear differentiation of classes and genera (Fig. 1, nucleotide tree, and Supplementary Fig. S2, protein tree) and were consistent with existing taxonomy and a tree reconstructed from 16S rRNA gene data (Fig. 2). The main difference between the tree generated from rMLST nucleotide data and that from the 16S rRNA gene data was the high level of resolution seen at the tips of the branches in the rMLST tree (Fig. 1). Both the 16S rRNA tree and the rMLST tree had uncertainty in the deep branches, and there was little phylogenetic signal below the level of class. Using rMLST data from the 144 isolates belonging to the class Bacilli enabled the relationships of related genera to be examined with clonalframe (Fig. 3). With this analysis, the available genomes belonging to the genus *Bacillus* formed three clades as distinct from each other as from *Listeria*. *Bacillus anthracis*, *Bacillus cereus* and *Bacillus weihenstephenensis* formed one clade; *Bacillus licheniformis*, *Bacillus amyloliquefaciens*, *Bacillus subtilis* and *Bacillus pumilus* another; and *Bacillus halodurans*, *Bacillus pseudofirmus* and *Bacillus clausii* the third (Fig. 3).

**Fig. 4.**
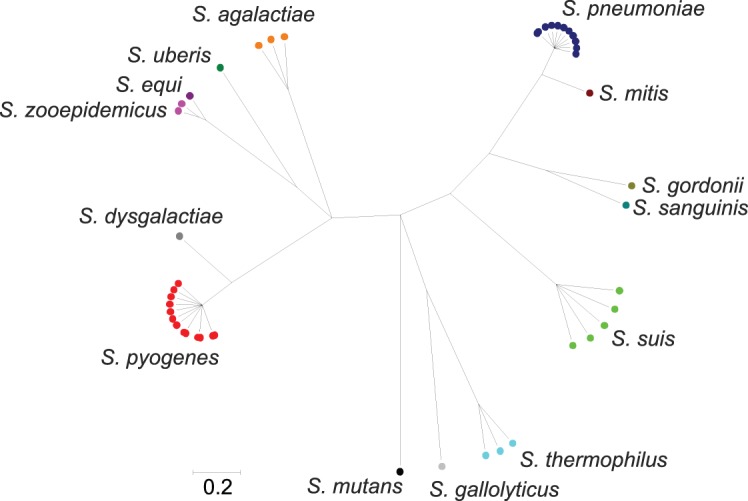
clonalframe tree of the genus *Streptococcus*. Three independent, converged runs were merged and a 95 % consensus tree generated. Only finished genomes with 53 ribosomal protein genes identified and tagged were included in the analysis (*n* = 45).

**Fig. 5.**
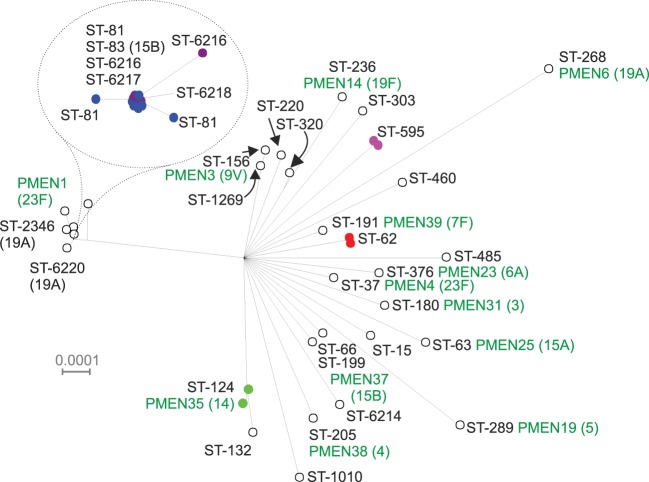
Split decomposition of concatenated ribosomal protein genes from *S. pneumoniae* isolates (*n* = 57). The *rpmG* gene was not included, since there appear to be three loci within the *S. pneumoniae* genome that can exhibit different *rpmG* alleles. The different PMEN clones within the dataset are clearly resolved and the heavily represented PMEN1 group centred around ST-81 shows sub-sequence type resolution.

Within the genus *Streptococcus*, which had a large number of genomes available, rMLST was able to differentiate distinct groups (Figs 3 and 4), largely corresponding to existing species designations. The 29 *S. pneumoniae* genomes downloaded from the IMG database (Markowitz *et al.*, 2010) were supplemented with assemblies generated from Illumina data for 28 genomes deposited in the EBI sequence read archive as part of a clinical study of the PMEN1 [Spain (23F) −1] clone (Croucher *et al.*, 2011). Split decomposition analysis of the concatenated rMLST loci demonstrated a high level of resolution within this single species, not only resolving individual PMEN clones but also resolving at a sub-sequence-type level within the PMEN1 group (Fig. 5). The rMLST loci vary considerably in length and discriminatory power both among loci and among taxonomic classes for specific loci (Supplementary Tables S2 and S3). Inspection of synonymous and non-synonymous substitutions within classes provided no significant evidence of positive selection. For any given species, the number of unique alleles was different among loci and is a consequence of the population structure of the organism. Monomorphic species such as *B. anthracis* and *Y. pestis* have very low sequence diversity, which is not the case for the majority of other bacteria. As an example of the former, relatively little discrimination was observed with the *Y. pestis* genomes in the database (Supplementary Fig. S3). Compared against a published phylogeny using 1364 single-nucleotide polymorphisms (SNPs) (Morelli *et al.*, 2010), rMLST could differentiate isolates belonging to the early branching 0.PE2 (Pestoides F) and 0.PE3 (Angola) populations.

### Congruence

The rMLST groupings generated were independent of the clustering algorithm used, whether it accounted for recombination, e.g. clonalframe, or not, indicating a strong clonal signal that was robust to horizontal genetic exchange. This was formally tested by a congruence test of 10 *rps* loci, chosen to be roughly equivalent to complete coding sequence lengths of between 400 and 1100 bp, within the class Bacilli (*n* = 144). This showed that although the topologies among trees drawn with single-locus data were significantly different from each other, these differences were comparatively small (Supplementary Tables S4 and S5), suggesting that phylogeny was largely conserved among these loci. Congruence was better among rMLST loci than among rMLST loci and random trees representing the range of topologies that would be expected if the loci were undergoing high levels of horizontal genetic exchange.

## Discussion

High-resolution bacterial characterization is of great importance to all areas of microbiology, but is particularly important for pathogenic bacteria, where rapid, precise identification of a disease-causing bacterium is often of high clinical or public health importance (van Belkum *et al.*, 2007). Pathogens of humans and their domesticated animals and plants have emerged many times from essentially all parts of the domain (Dethlefsen *et al.*, 2007; Ciccarelli *et al.*, 2006), and therefore a general approach that works for clinical specimens will be applicable to all members of the domain. Nucleotide sequence data, especially of protein-encoding genes, have a number of advantages in both taxonomic and typing schemes, as they are high-resolution, reproducible and portable, and can be analysed with a variety of evolutionary and population genetic approaches (Maiden *et al.*, 1998). The development of a unified scheme has, however, been hindered by the extensive diversity of the bacterial domain with members, most yet to be cultured (Rappé & Giovannoni, 2003), found in virtually all known biological niches and participating in the majority of biochemical processes described to date (Krieg *et al.*, 2005); consequently, few core metabolic genes that can be targeted in a universal genealogy are shared by all bacteria.

The widespread occurrence of horizontal genetic exchange among diverse members of the bacterial domain provides a further obstacle to phylogeny-based bacterial characterization, as different loci within the same genome can have widely different evolutionary histories (Holmes *et al.*, 1999). This is especially true of the accessory genome – the genetic material that is not present in all members of the group. Hence most approaches to establishing the relationships among bacteria have concentrated on using multiple core-genome loci which are under stabilizing selection. What constitutes a ‘core genome’ is, however, difficult to define, and differs among different bacterial groups: this is why separate MLST schemes are required even for quite closely related bacteria (Maiden, 2006). Sequence variation in the core genome is also subject to horizontal genetic exchange, leading to incongruent phylogenies (Feil *et al.*, 2001), and as incongruence can also be generated by the saturation of variable sites, the simple act of including more sequences does not necessarily generate a reliable phylogeny (Jeffroy *et al.*, 2006). The generation of robust phylogenetic groupings therefore requires the appropriate choice of multiple genetic loci.

The 53 *rps* genes, which are shared and functionally conserved amongst all members of the domain, and indeed across the three domains of life (Roberts *et al.*, 2008; Matte-Tailliez *et al.*, 2002), are among the few candidate loci that can be targeted by a combined taxonomic and typing system for all bacteria. Their distribution around the bacterial chromosome (Fujita *et al.*, 1998) and the increasing understanding of their structure–function relationships (Bashan & Yonath, 2008) represent further advantages. Using these loci for combined taxonomy and typing is an extension of the highly successful 16S rRNA gene approach (Woese, 1987), and it is worthy of note that previous attempts to define a set of core genes shared among all bacterial genomes have identified many, but not all, of the ribosomal subunit genes (Wu *et al.*, 2008, 2009; Ciccarelli *et al.*, 2006; Teeling & Gloeckner, 2006). This is because there is appreciable variation at a number of the *rps* loci across the whole domain (Hansmann & Martin, 2000); however, it is the inclusion of the more variable genes that gives the subspecies resolution achieved by rMLST, and that makes it especially powerful as a universal tool. The identification, indexing and curation of the *rps* genes across the domain described here represent a first step in an ongoing process, equivalent to that currently undertaken for MLST schemes (Maiden, 2006). The BIGSdb platform can accommodate this effort, as any number of curators, each with defined editing privileges over particular schemes, can be assigned, and the system maintains logs of changes made to annotation or locus definitions (Jolley & Maiden, 2010). In addition the rMLST scheme can co-exist with any number of other schemes incorporating the same or different loci, enabling easy cross-referencing of the various schemes and the combination of, for example, rMLST with conventional MLST, antigen fine typing, or antibiotic resistance deduction from nucleotide sequences. Processing of whole-genome data using rMLST and the BIGSdb platform is rapid – *de novo* sequence assembly of short-read data for most bacterial genomes can be achieved in 1–2 h using current modest computing resources, and this will improve as longer read lengths become routine. Uploading these to the database and allele identification takes about a minute in total to obtain a complete rMLST profile. Provided comparable isolates are present within the database, accurate typing for most species can then be performed instantaneously.

There remains much debate about the bacterial species concept (Fraser *et al.*, 2009), and indeed whether bacterial ‘species’ meaningfully exist (Doolittle & Zhaxybayeva, 2009). As increasing quantities of data are accumulated, it is becoming clear that the majority of bacterial types, assigned variously to groups, genera, species, etc. on the basis of phenotypic properties, mostly represent clusters of sequence diversity, with areas of vacant sequence space between them (Fraser *et al.*, 2009). This uneven landscape of bacterial diversity, however, includes some clusters that remain distinct from their close relatives and are of recent evolutionary divergence (Richter & Rosselló-Móra, 2009). These small differences can have marked and stable phenotypic consequences, as exemplified by single-clone pathogens such as *B. anthracis*, *Y. pestis* and *Neisseria gonorrhoeae*. These pathogens have emerged recently, and although genetically very similar to non-pathogenic members of the same genus, their stable phenotypic properties render them biologically distinct from their close relatives and warrant distinct names (Achtman, 2008). Ultimately, the work of systematic microbiology will continue to be the association of particular phenotypes with given genetic types, and this requires many factors to be taken into account, with the assignment of clusters to named groups on the basis of sequence divergence alone unlikely to be completely satisfactory (Moore *et al.*, 2010). The low number of genomes currently available for type strains is also problematic for robust species identification, although this issue will naturally improve over time. The clusters of sequences identified with rMLST data provide a universally applicable dataset around which descriptions of bacterial diversity can be assembled, complementing methods such as whole-genome hybridization (Tindall *et al.*, 2010), which is not a practical approach for certain data, such as those obtained in metagenomic studies (Stackebrandt *et al.*, 2002).

Here we show the resolution that rMLST can achieve from the whole-domain level down to the subspecies level for the *S. pneumoniae* PMEN1 clone. Independently of the clustering algorithm used, rMLST data assigned bacteria to groups that were in agreement with the current family, order and genus designations (Figs 1 and 2, and Supplementary Fig. S2). Further, rMLST replicated the inter- and intra-species relationships of streptococcal isolates established by MLST and MLSA (Bishop *et al.*, 2009; Do *et al.*, 2009) approaches (Figs 3 and 4) and these groups were robust to the high levels of genetic exchange seen in these bacteria. Within *S. pneumoniae*, isolates belonging or closely related to the major disease-associated clones, as identified by the PMEN (McGee *et al.*, 2001), were easily distinguished, with a high degree of resolution among isolates within the PMEN-1 clone (Croucher *et al.*, 2011), at least equivalent to that obtained with MLST (Fig. 5). Thus in an era of expanding sequencing capacity, rMLST represents a portable and efficient means of interpreting whole-genome data for clinical and other purposes, enabling a recently assembled draft sequence to be assigned to strain level rapidly and unambiguously. Not every locus will be required for every application, and the number of loci to include in an analysis will depend on the level of resolution required. For species identification, the complete sequence of any single locus is likely to be sufficient, whereas for strain-level typing, a subset will be necessary depending on the species. For monomorphic species such as *Y. pestis* and *B. anthracis*, all *rps* loci would be used, although this will be insufficient for fine-resolution typing (Supplementary Fig. S3); however, rMLST is implemented through BIGSdb which is entirely compatible with other finetyping schemes such as variable-number tandem repeats (VNTR) or canonical SNP analysis (Keim *et al.*, 2004). Conversely for highly recombinagenic species such as *Helicobacter pylori*, approximately seven loci would give a similar resolution to standard MLST (Supplementary Table S2). Within particular species some loci are more discriminatory than others, but the discriminatory loci are not the same throughout the domain. Phylogenetic analysis of distantly related members of the domain may require use of protein sequences to minimize the generation of misleading relationships due to multiple substitutions and branch attraction by extreme AT- or GC-rich genomes.

The gene-by-gene approach to population genomics adopted here has a number of advantages over methods of multiple genome comparison that rely on whole-genome alignment and multiple pairwise comparisons (Carver *et al.*, 2005; Darling *et al.*, 2010), or the identification of informative SNPs by mapping against a related reference genome (Harris *et al.*, 2010). Importantly, the approach does not require closed reference genomes and is highly scalable, with the time taken to analyse genomes increasing linearly with the number of genomes and loci included. Furthermore, the reanalysis of existing allele designations is not required as further data are added, and since the units of analysis are single genes, genomic data can be analysed irrespective of the size of the assembled contiguous sequences, or contigs, provided most loci are encompassed within a single contig, which we have shown is usually the case for protein-encoding genes in the data analysed here. The approach is also robust with respect to missing data in incomplete datasets, which makes it particularly suitable for the analysis of data generated with the current generation of parallel sequencing technologies, which have short read lengths, resulting in genome assemblies comprising multiple contigs. As allele identification is performed by the comparison of a single gene from an isolate against the entire known diversity of that locus, the method can be used to analyse highly divergent isolates, including those from different species or genera. The approach can be applied to any sequence string, either nucleotide or peptide, and multiple strings (loci) can be flexibly grouped into any number of ‘schemes’, each of which is equivalent to an MLST scheme (Jolley & Maiden, 2010).

In conclusion, the 53 *rps* genes represent a core genome that is sufficiently conserved across the whole domain to form the basis of a combined taxonomic and typing scheme, yet contains sufficient diversity for high-resolution isolate characterization. The relationships among isolates obtained with rMLST data are independent of the clustering algorithm used and robust to horizontal genetic exchange. Additional advantages of using the *rps* genes include their distribution in several chromosomal locations and the fact that they are protein-encoding, enabling the interpretation of their diversity with a variety of evolutionary models. Furthermore, the ribosome occupies the interface between genotype and phenotype that is a required focus of microbiology in the post-genomic era of research. For many or most clinical purposes, rMLST data will provide not only definitive but complete typing information, although in the BIGSdb system these data can be readily supplemented with complementary typing schemes, if further resolution is required. The rMLST approach is a natural extension of the very successful use of the 16S rRNA gene for microbial taxonomy (Woese *et al.*, 1990), with the advantages of being multilocus and containing higher levels of discrimination. The adoption of this approach is not a panacea that will in itself resolve the many issues of nomenclature and typing that are inherent in the cataloguing of the extensive diversity of the bacterial domain; however, as was the case with MLST at the bacterial species and genus level, rMLST will provide a universal reference point that can complement existing methods to assist in the rational interpretation of patterns of bacterial diversity.
